# Supplementary material for: ORP1L mediated PI(4)P signaling at ER-lysosome-mitochondrion three-way contact contributes to mitochondrial division
Source: Nat Commun. 2021 Sep 9;12:5354. doi: 10.1038/s41467-021-25621-4 (PMC8429648; doi:10.1038/s41467-021-25621-4)
Supplement: Supplementary file 1 — Supplementary Information [file 41467_2021_25621_MOESM1_ESM.pdf]

Supplementary information

ORP1L mediated PI(4)P signaling at ER-lysosome-mitochondrion three-way contact contributes to mitochondrial division

Authors and affiliations:

Maxime Boutry[1], Peter K. Kim[1,2]*

1: Cell Biology Program, Hospital for Sick Children, Peter Gilgan Centre for Research and Learning, Toronto, Ontario M5G0A4, Canada

2: Department of Biochemistry, University of Toronto, Toronto, Ontario M5S1A8, Canada

***** Corresponding author: Peter K. Kim

Peter Gilgan Centre for Research & Learning

The Hospital for Sick Children

686 Bay Street, 19-9800

Toronto, Ontario M5G0A4, Canada

Tel: 416-813-5983

Fax : 416-813-5028

E-mail: pkim@sickkids.ca (P.K.K)

Supplementary Figure 1

a

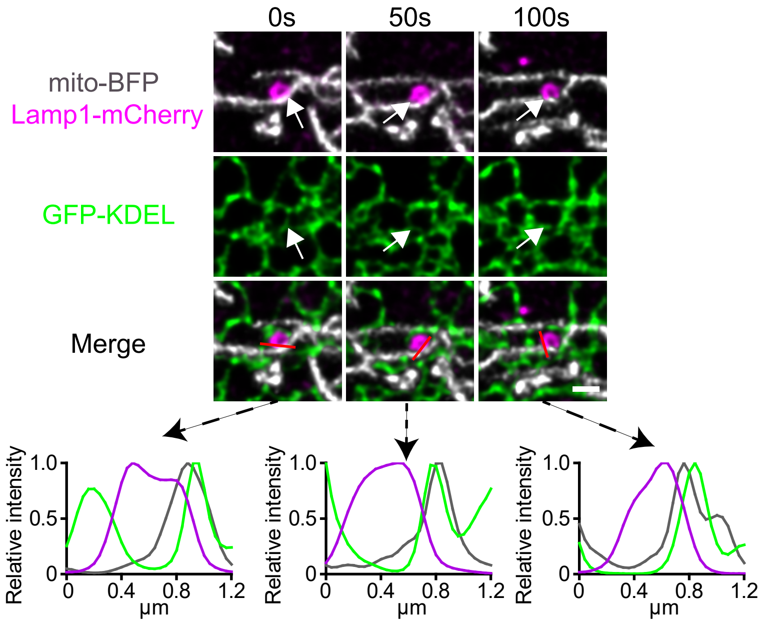

b

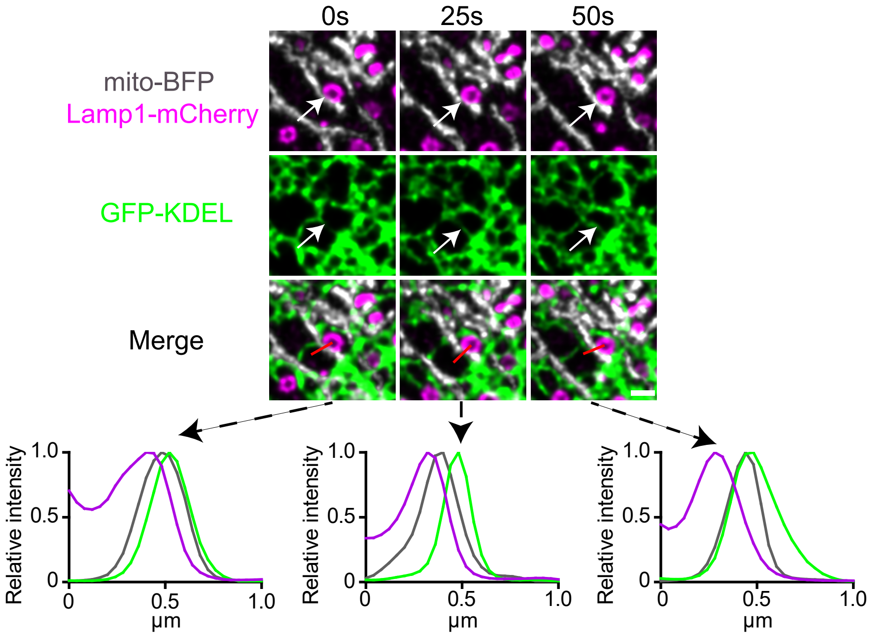

c

3-way contacts

Outcome	n = 100 events
Untethering	86 %
Remain tethered	11 %
Mitochondrial division	3 %

n=20 cells

Supplementary Figure 1. Three-way contacts between the ER, lysosomes and mitochondria are stable and can be observed at mitochondrial division sites.

a, b Two Representative images of lysosome-mitochondria contacts that are stably marked by the ER for at least 100 (**a**) and 50 seconds (**b**), respectively. White arrows indicate the lysosome-mitochondria contact at different time points. Line-scan analysis of relative fluorescence intensity from the red lines are shown. Scale bar: 1µm. **c** The outcome of 100 randomly chosen pre-existing three-way contacts between the ER, lysosomes and mitochondria from 20 HeLa cells from two independent experiments was evaluated and expressed in percentage (5 random three-way contacts per cell were monitored). Most 3-way contacts untethered (n=86/100) overtime and a small proportion (n=3/100) engaged in the division of mitochondria while the others (n=11/100) remained tethered during the complete duration of the acquisition.

Supplementary Figure 2

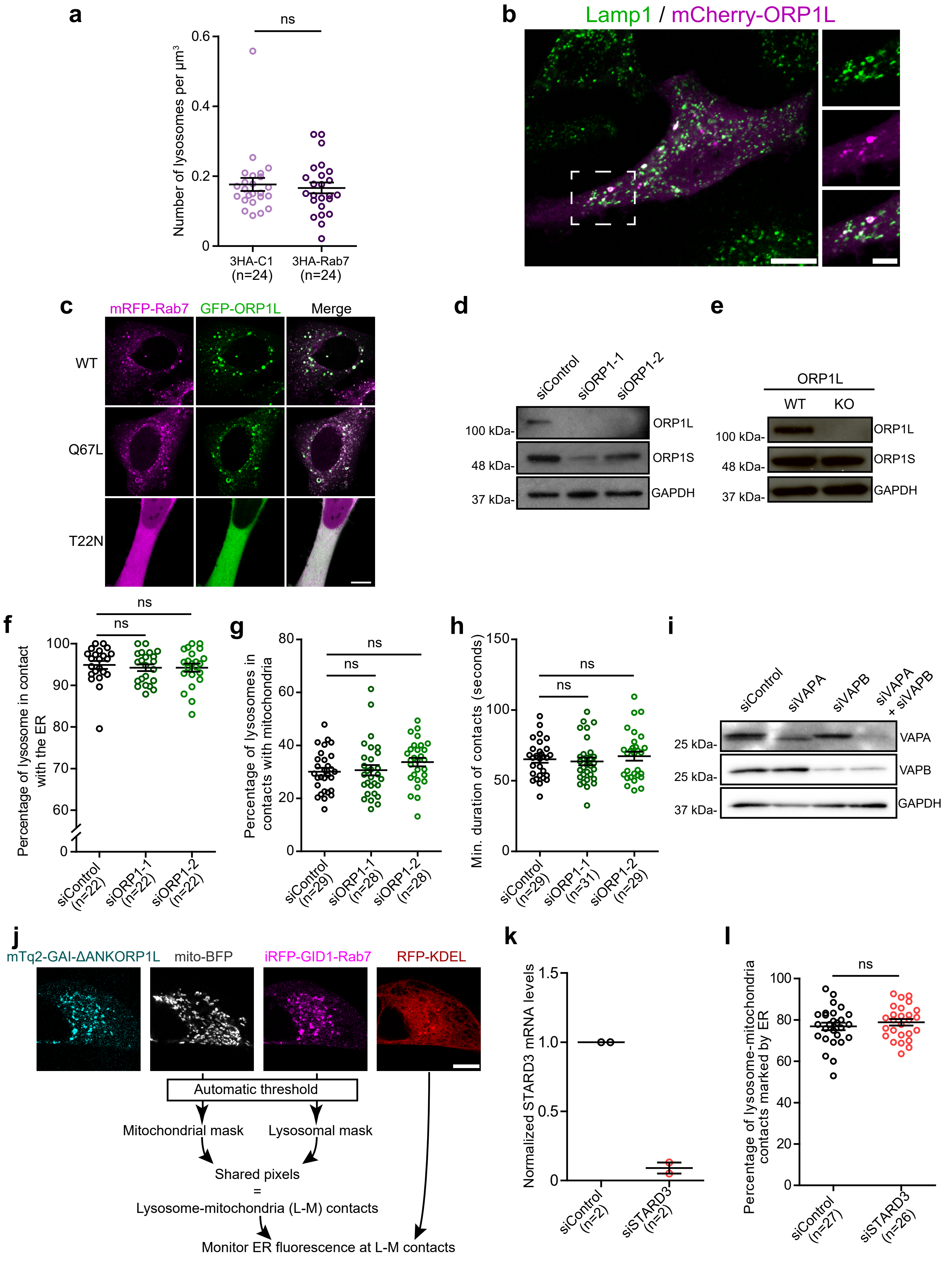

Supplementary Figure 2. A Rab7-ORP1L-VAPs interaction regulates the presence of the ER at lysosome-mitochondria contacts in HeLa cells.

a Number of lysosomes in cells overexpressing the empty vector 3HA-c1 or 3HA-Rab7. The graphs show the mean ± SEM, cells from three independent experiments. Two-sided unpaired t-test, ns = non-significant, p=06830. **b** Representative image of a cell expressing mCherry-ORP1L and immunostained for Lamp1. Scale bars: 10µm and 5µm (inset). **c** Representative images of cells co-expressing GFP-ORP1L and WT, Q67L or T22N mRFP-Rab7. Scale bar: 10µm. **d, e** Western blot images (**d**) showing the downregulation of ORP1L and ORP1S upon transfection by siRNA targeting specifically ORP1; (**e**) confirming the absence of ORP1L, but normal ORP1S levels, in ORP1L KO HeLa cells compared to control cells. GAPDH was used as a loading control. Images are representative of two independent western blots. **f** Quantification of the percentage of lysosome in contact with the ER in cells transfected with siRNA targeting ORP1 and expressing Lamp1-mCherry and GFP-KDEL. The graphs show the mean ± SEM, cells from two independent experiments. One-way ANOVA with Dunnett's Multiple Comparison Test, ns = non-significant, p=08268 (siORP1-1) and p=0.8290 (siORP1-2). **g, h** (**g**) Percentage of lysosomes in contact with mitochondria and (**h**) minimum duration of these contacts in cells treated with the indicated siRNAs. Cells from two independent experiments. One-way ANOVA with Dunnett's Multiple Comparison Test, ns = non-significant, (**g**) p=0.9602 (siORP1-1) and p=0.2275 (siORP1-2) and (**h**) p=0.8962 (siORP1-1) and p=0.8099 (siORP1-2). **i** Western blot images showing the downregulation of VAPs upon transfection by siRNA targeting specifically VAPA or VAPB. GAPDH was used as a loading control. Images are representative of two independent western blots. **j** Schematic representation of the methodology used to three-way contacts in Fig 3n. Scale bar: 10µm. **k** Normalized STARD3 mRNA levels in cells transfected by indicated siRNAs measured by RT-qPCR. The graphs show the mean ± SEM, n= 2 independent experiments. **l** Quantification of the percentage of lysosome-mitochondria contacts marked by the ER in cells treated with indicated siRNAs expressing Lamp1-mCherry GFP-KDEL and mito-BFP. The graphs show the mean ± SEM, cells from two independent experiments. Two-sided unpaired t-test, ns = non-significant, p=04123.

Supplementary Figure 3

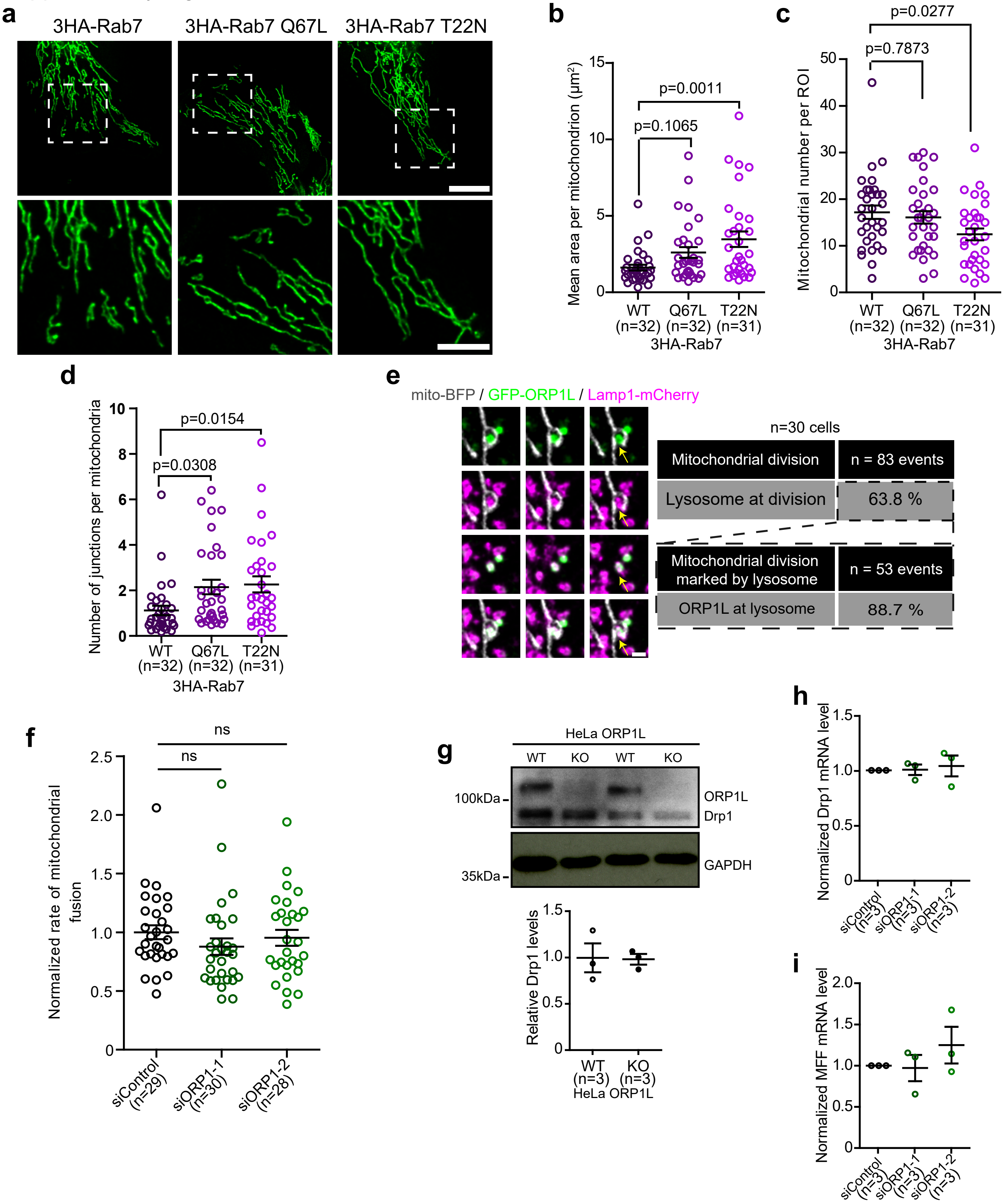

Supplementary Figure 3. The Rab7-ORP1L-VAPs interaction regulates mitochondrial division.

a Representative maximum projection images of mitochondrial morphology in HeLa cells overexpressing the wild-type, the Q67L or T22N mutant 3HA tagged Rab7 and mito-GFP. Scale bars: 10µm and 5µm (inset). **b-d** Mitochondrial morphology was quantified for (**b**) mean area per mitochondrion, (**c**) mitochondrial number per region of interest (ROI) and (**d**) number of junctions per mitochondria. Cells from three independent experiments. One-way ANOVA with Dunnett's Multiple Comparison Test. The graphs show the mean ± SEM. **e** Live-cell imaging of HeLa cells expressing mito-BFP, Lamp1-mCherry and GFP-ORP1L. Yellow arrows indicate a GFP-ORP1L positive lysosome that is recruited to a mitochondrial fission event. Scale bar: 1 µm. The table show the percentage of mitochondrial division events marked by lysosomes (n = 30 cells) and the percentage of lysosomes at mitochondrial division events that were positive for GFP-ORP1L. **f** Normalized rate of mitochondrial fusion in HeLa cells treated with the indicated siRNAs and expressing mito-GFP. Cells from three independent experiments. One-way ANOVA, with Dunnett's Multiple Comparison Test. ns = non statistically significant: (**f**) p=0.3244 and (**g**) p=0.8399. The graphs show the mean ± SEM. **g** Western blot image showing ORP1L and Drp1 levels in ORP1L WT and KO HeLa cells. The expression of GAPDH was used as a loading control. The Image shows three technical replicates loaded on the same blot and the densitometry analysis of the blot. **h-i** Normalized (**h**) Drp1 mRNA levels and (**i**) MFF mRNA levels in cells transfected by two siRNA targeting specifically ORP1 or a control siRNA measured by RT-qPCR. The graphs show the mean ± SEM, n= 3 independent experiments.

Supplementary Figure 4

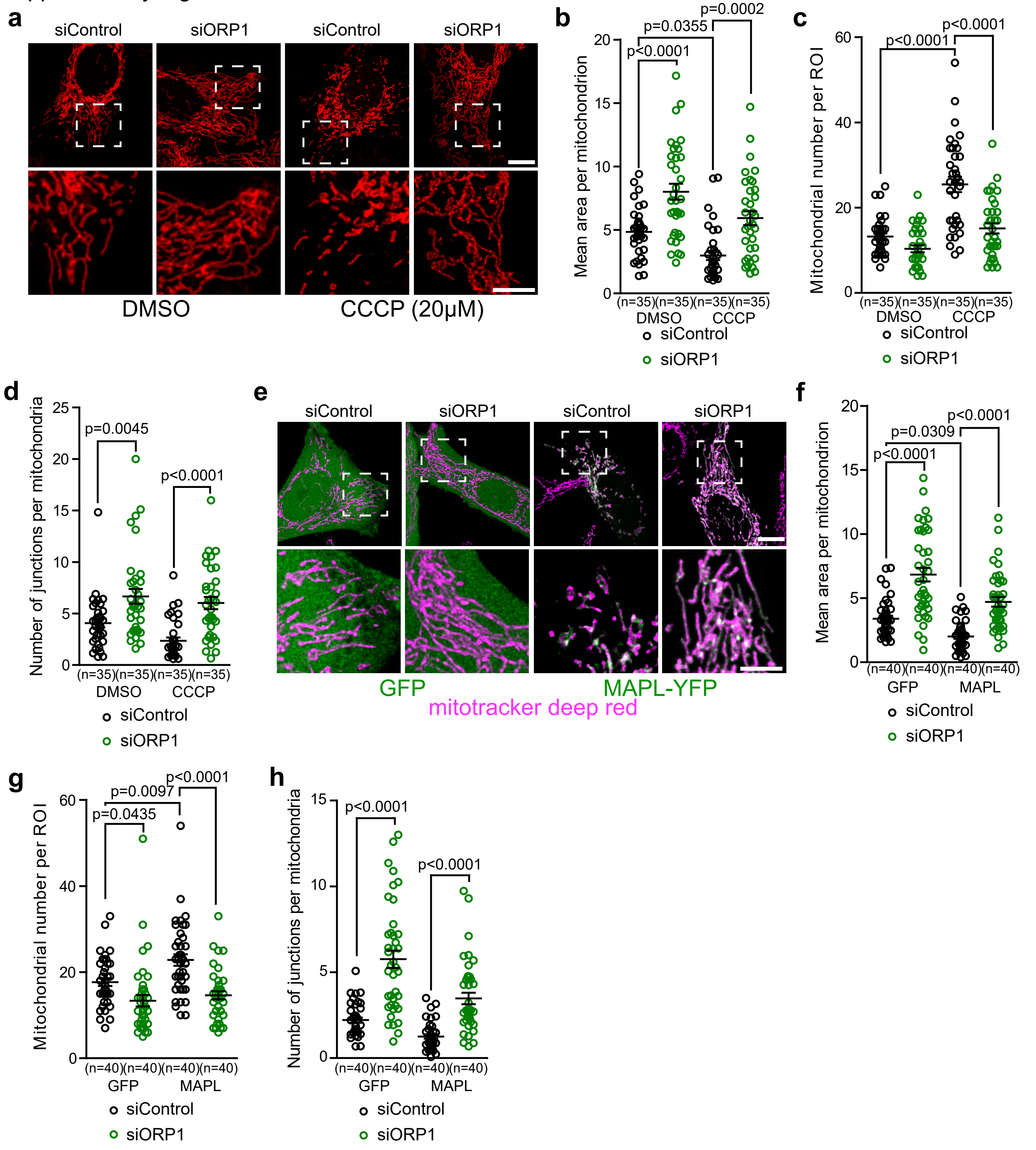

Supplementary Figure 4. Loss of ORP1L protects from Drp1 dependent stimulated mitochondrial division.

a Representative maximum projection images of mitochondrial morphology in HeLa cells overexpressing mApple-TOM20 and treated with the indicated siRNA. Cells were treated with CCCP (20µM) for 30 minutes to stimulate mitochondrial division in a Drp1 dependent manner. Scale bars: 10µm and 5µm (inset). **b-d** Mitochondrial morphology was quantified for (**b**) mean area per mitochondrion, (**c**) mitochondrial number per region of interest (ROI) and (**d**) number of junctions per mitochondria. Cells from three independent experiments. Two-way ANOVA with Tukey's Multiple Comparison Test. The graphs show the mean ± SEM. **e** Representative maximum projection images of mitochondrial morphology in HeLa cells overexpressing GFP or MAPL-YFP (which stimulates Drp1 dependent mitochondrial division) and treated with the indicated siRNA. Mitotracker was used to visualize mitochondria. Scale bars: 10µm and 5µm (inset). **f-h** Mitochondrial morphology was quantified for (**f**) mean area per mitochondrion, (**g**) mitochondrial number per region of interest (ROI) and (**h**) number of junctions per mitochondria. Cells from three independent experiments. Two-way ANOVA with Tukey's Multiple Comparison Test. The graphs show the mean ± SEM.

Supplementary Figure 5

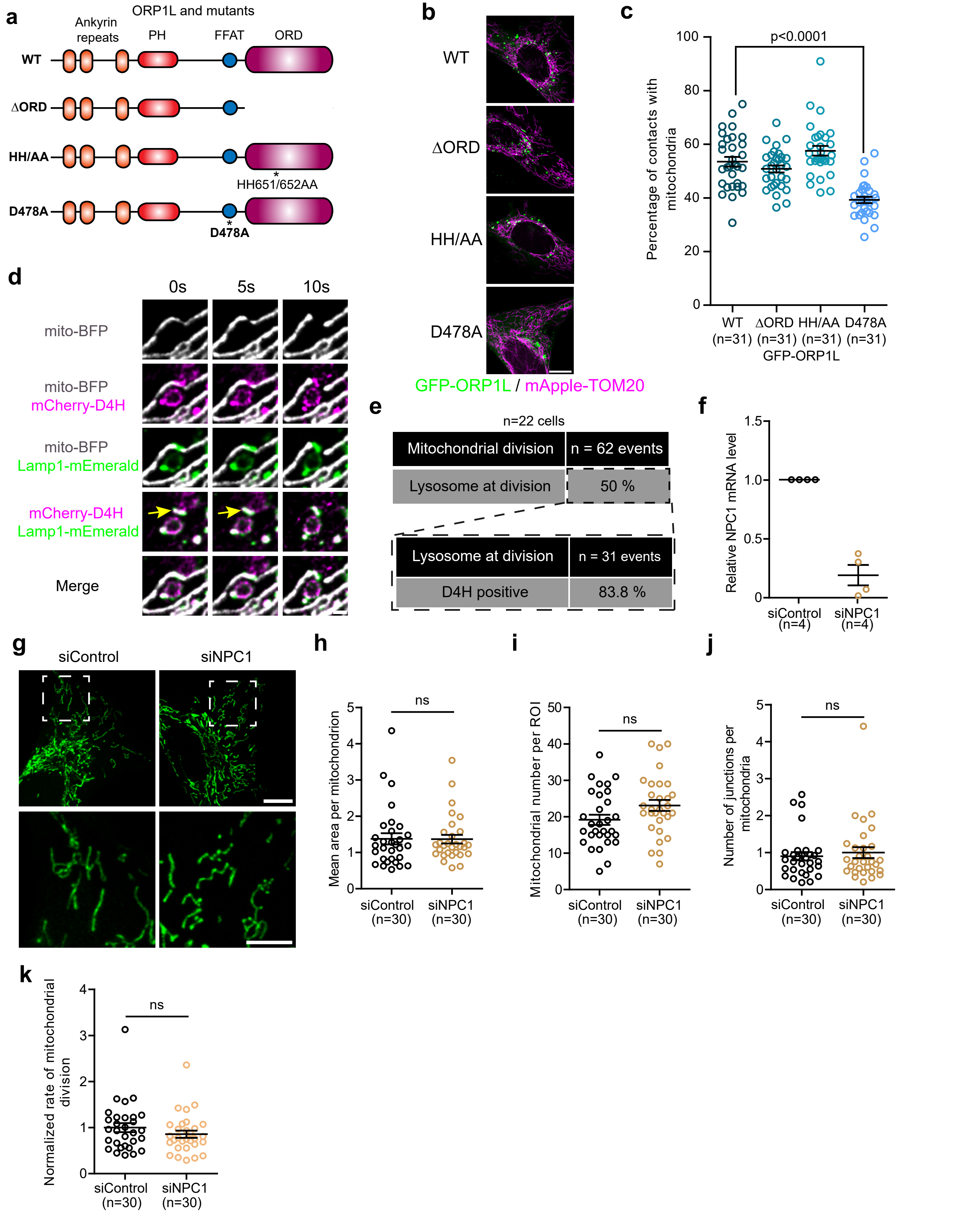

Supplementary Figure 5. ORP1L mutants used in the study and NPC1 downregulation does not affect mitochondrial division.

a Schematic representation of wild-type ORP1L and ORP1L mutants used in the study. Domains are not to scale. Mutations are indicated by *. **b** Representative images of HeLa cells expressing WT and mutants GFP-ORP1L constructs and mApple-TOM20. Scale bar: 10µm. **c** Percentage of ORP1L WT and mutants' positive lysosomes in contact with mitochondria in HeLa cells expressing mApple-TOM20. The graphs show the mean ± SEM, cells from four independent experiments. One-way ANOVA with Dunnett's Multiple Comparison Test. **d** Live-cell imaging of HeLa cells expressing mito-BFP, Lamp1-mEmerald and the cholesterol probe mCherry-D4H. Yellow arrows indicate a lysosome positive for the mCherry-D4H probe and that is recruited to a mitochondrial division event. Scale bar: 1 µm. **e** Percentage of mitochondrial division events marked by lysosomes (22 cells from two independent experiments) and the percentage of lysosomes at mitochondrial division events that were positive for the cholesterol probe mCherry-D4H. **f** Normalized NPC1 mRNA levels in cells treated by a siRNA targeting specifically NPC1 or a control siRNA measured by RT-qPCR. The graphs show the mean ± SEM, n= 4 independent experiments. **g** Representative maximum projection images of mitochondrial morphology in HeLa cells treated with the indicated siRNA. mito-GFP was used as a mitochondrial marker. Scale bars: 10µm and 5µm (inset). **h-j** Mitochondrial morphology was quantified for (**h**) mean area per mitochondrion, (**i**) mitochondrial number per region of interest (ROI) and (**j**) number of junctions per mitochondria. Cells from three independent experiments. Two-sided unpaired t-test, ns = non statistically significant: (**h**) p=0.9538, (**i**) p=0.0683 and (**j**) p=0.6092. The graphs show the mean ± SEM. **k** Normalized rate of mitochondrial division in HeLa cells treated with the indicated siRNAs and expressing mito-GFP. Cells from three independent experiments. Two-sided unpaired t-test, ns = non statistically significant: p=0.2551. The graphs show the mean ± SEM.

Supplementary Figure 6

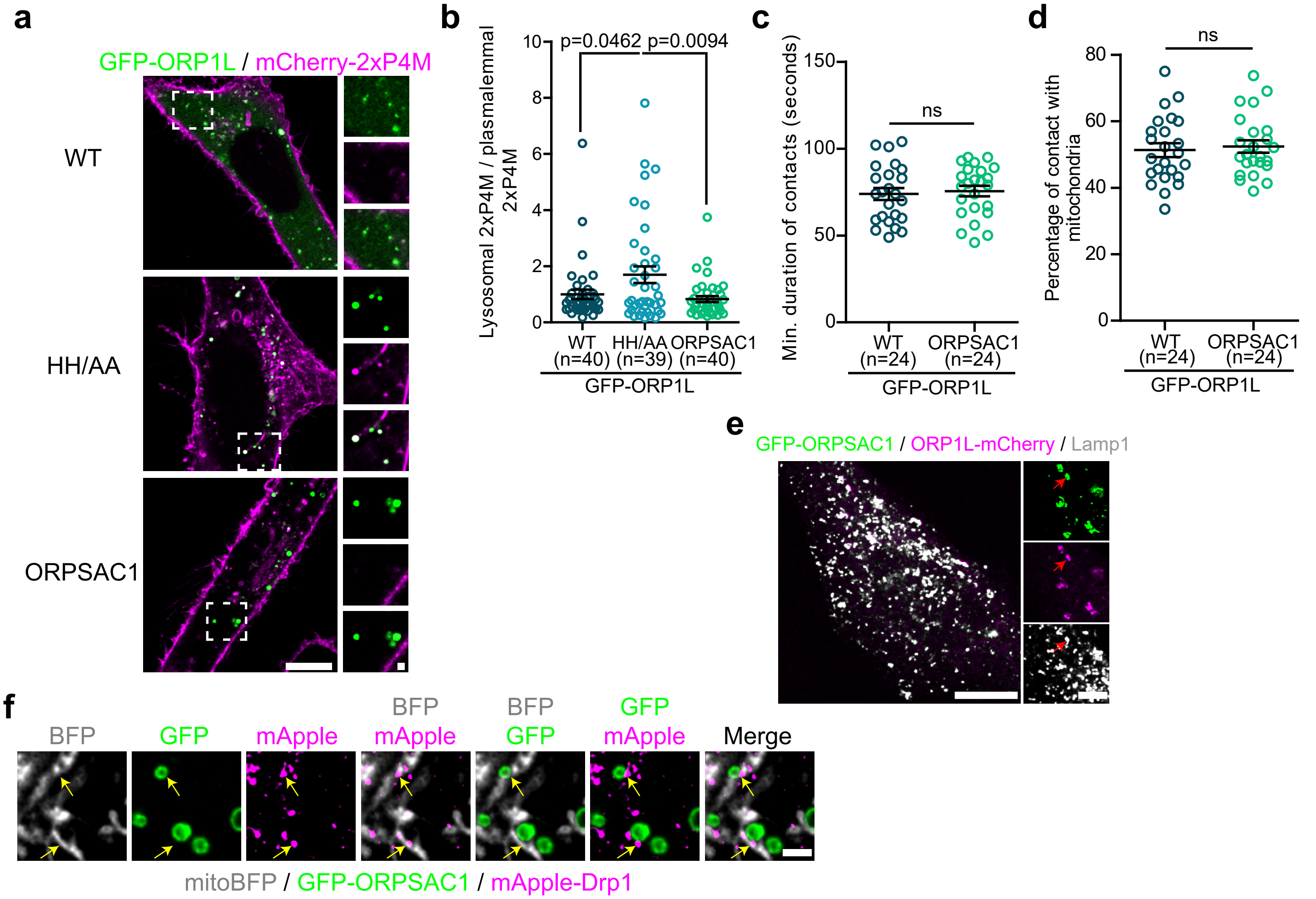

Supplementary Figure 6. Lysosomal PI(4)P is involved in mitochondrial division.

a Representative images of Hela cells expressing the PI(4)P probe mCherry-2xP4M and either WT, HH/AA GFP-ORP1L or GFP-ORPSAC1 constructs. Note that ORP1L WT and ORPSAC1 positive lysosomes show a weak colocalization with 2xP4M probe whereas the HH/AA ORP1L mutant positive lysosomes show a stronger colocalization. Scale bars: 10µm and 1µm (inset). **b** Quantification of the lysosomal levels of 2xP4M normalized by the plasmalemmal levels of the probe. The graphs show the mean ± SEM, cells from three independent experiments. One-way ANOVA with Tukey's Multiple Comparison Test. **c** Quantification of the minimum duration of GFP-ORP1L or GFP-ORPSAC1 positive lysosome-mitochondria contacts. mApple-TOM20 was used as a mitochondrial marker. The graphs show the mean ± SEM, cells from three independent experiments. Two-sided unpaired t-test, ns = non statistically significant: p=0.7165. **d** Quantification of the percentage of either GFP-ORP1L or GFP-ORPSAC1 positive lysosomes in contact with mitochondria. mApple-TOM20 was used as a mitochondrial marker. The graphs show the mean ± SEM, cells from three independent experiments. Two-sided unpaired t-test, ns = non statistically significant: p=0.6959. **e** Representative image of an HeLa cells expressing GFP-ORPSAC1, mCherry-ORP1L and immunostained with an anti-Lamp1 antibody. Scale bars: 10µm and 5µm (inset). **f** Live cell imaging of a GFP-ORPSAC1 positive lysosomes in contact with a mitochondrial constriction site marked by an mApple-Drp1 puncta. mito-BFP was used as a mitochondrial marker. Scale bar: 1µm.

Supplementary Figure 7

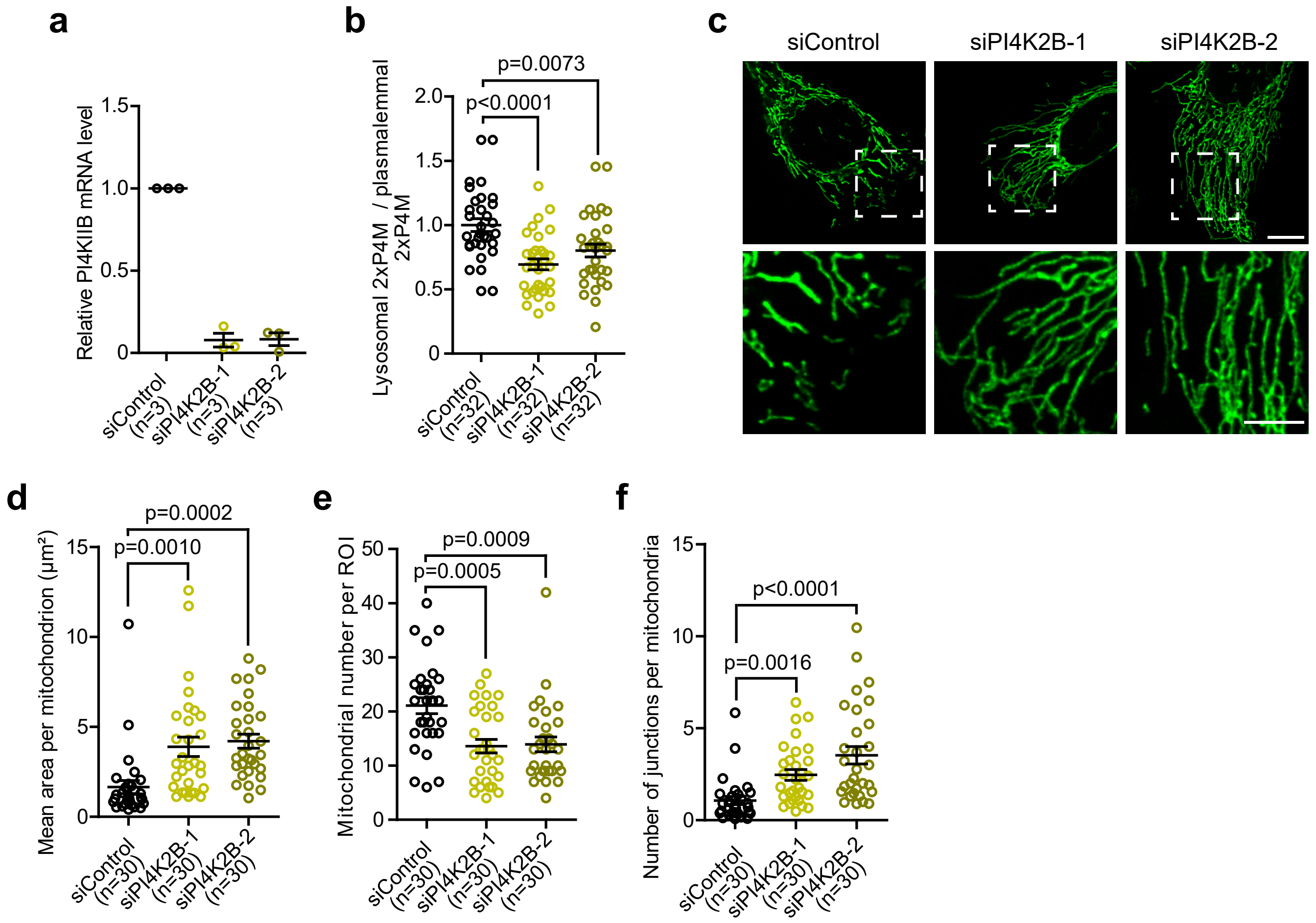

Supplementary Figure 7. Downregulation of the lysosomal localized PI4K2B alters mitochondrial morphology.

a Normalized PI4K2B mRNA levels in cells treated with the indicated siRNA and measured by RT-qPCR. The graphs show the mean ± SEM, n= 3 independent experiments. **b** Quantification of the lysosomal levels of 2xP4M normalized by the plasmalemmal levels of the probe in HeLa cells treated with the indicated siRNA. The graphs show the mean ± SEM, cells from three independent experiments. One-way ANOVA with Dunnett's Multiple Comparison Test. **c** Representative maximum projection images of mitochondrial morphology in HeLa cells treated with the indicated siRNA. mito-GFP was used as a mitochondrial marker. Note that treatment with siRNA targeting PI4K2B expression induce an elongation and hyperfusion of mitochondria as compared to the control siRNA Scale bars: 10µm and 5µm (inset). **d-f** Mitochondrial morphology was quantified for (**d**) mean area per mitochondrion, (**e**) mitochondrial number per region of interest (ROI) and (**f**) number of junctions per mitochondria. Cells from three independent experiments. One-way ANOVA with Dunnett's Multiple Comparison Test. The graphs show the mean ± SEM.

Supplementary Figure 8

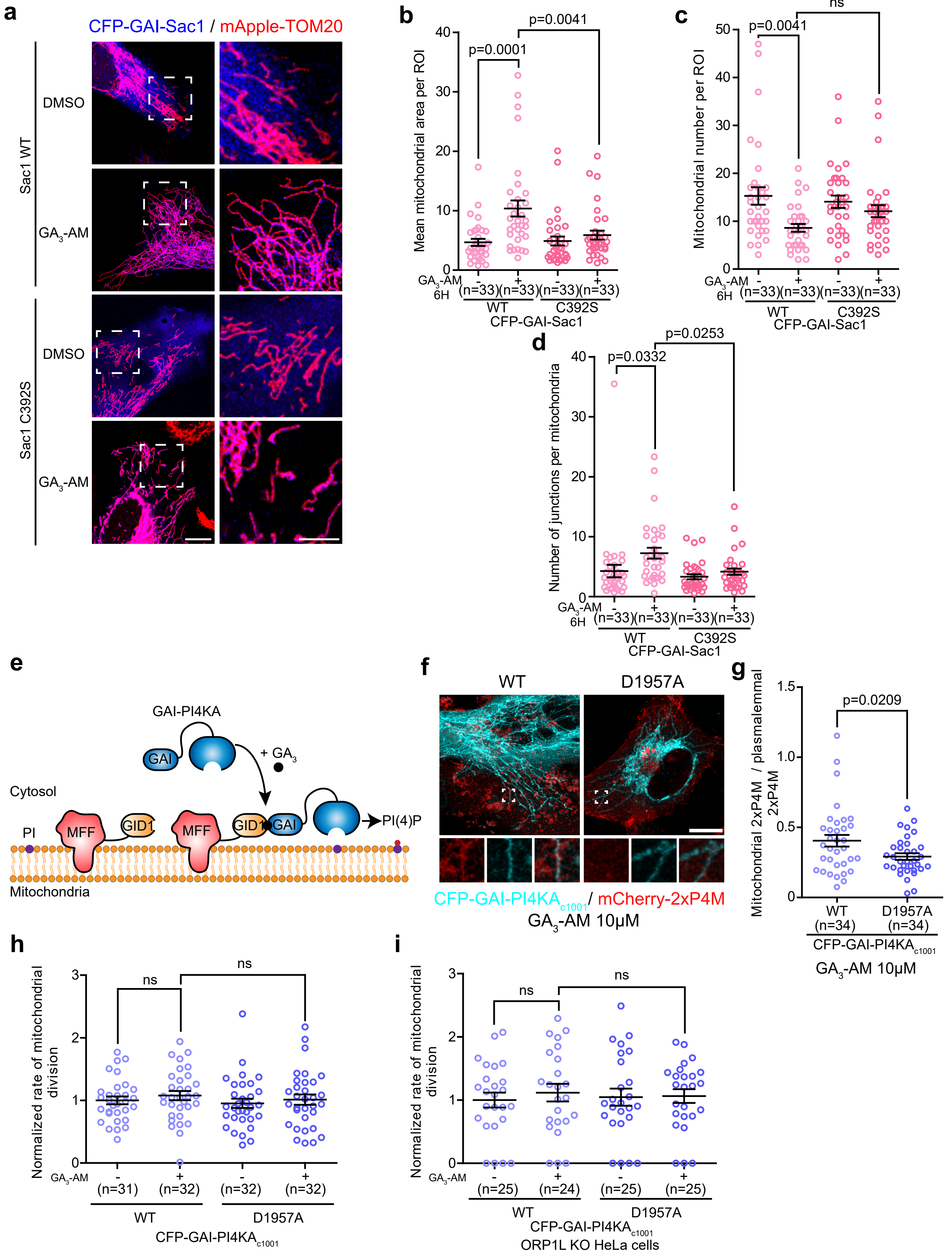

Supplementary Figure 8. Recruitment of Sac1 or PI4KAc1001 to mitochondria and their effect on mitochondrial division.

a Representative maximum projection images of mitochondrial morphology in HeLa cells overexpressing the wild-type CFP-GAI-Sac1 or the catalytic dead C392S mutant and iRFP-GID1-MFF (not imaged) before and after 6 hours of GA3-AM (10µM) treatment. Scale bars: 10µm and 5µm (inset). **b-d** Mitochondrial morphology was quantified for (**b**) mean area per mitochondrion, (**c**) mitochondrial number per region of interest (ROI) and (**d**) number of junctions per mitochondria in HeLa cells expressing wild-type CFP-GAI-Sac1 or the catalytic inactive C392S mutant, iRFP-GID1-MFF and mApple-TOM20, after 6 hours of GA_3-AM (10µM) treatment (DMSO was used as a control). Cells from three independent experiments. Two-way ANOVA with Sidak's multiple comparisons test. ns = non-significant: p=0.2647. The graphs show the mean ± SEM. **e** Soluble GAI-PI4KAc1001 (catalytic domain of PI4KA) can be recruited to mitochondrial fission site using GID1-MFF upon GA_3-AM treatment, leading to production of PI(4)P at the mitochondrial division site. **f, g** (**f**) Representative images of HeLa cells expressing CFP-GAI-PI4KAc1001 or the catalytic dead D1957A mutant, iRFP-GID1-Rab7 and mCherry-2xP4M after GA_3-AM treatment. Scale bars: 10µm and 1µm (inset). (**g**) Quantification of the mitochondrial levels, normalized by the plasmalemmal levels, of 2xP4M. The graphs show the mean ± SEM, cells from three independent experiments. Two-sided unpaired t-test. **h, i** Normalized rate of mitochondrial division before and after GA_3-AM (10µM) treatment in (**h**) HeLa cells or (**i**) ORP1L KO HeLa cells overexpressing the wild-type CFP-GAI-PI4KAc1001 or the inactive D1957A mutant, iRFP-GID1-MFF and mApple-TOM20. When treated with GA_3-AM (10µM) cells were imaged between 5 and 25 minutes of treatment. Cells from three independent experiments. Two-way ANOVA, Sidak's multiple comparisons test, ns = non-significant: (h) p=0.8797 and p=0.9249, (i) p=0.9138 and p=0.9904. The graphs show the mean ± SEM.

Supplementary Table 1. List of primers used for cloning. (see Plasmids section in the Methods of the manuscript).

All primers have been custom synthesized and purchased from Idt (Integrated DNA technologies).

Construct	Insert or Vector		Sequence
3HA-Rab7 WT, Q67L and T22N	Insert = Rab7 WT, Q67L or T22N	Forward	tagctctcgagccATGACCTCTAGGAAGAAAGTGTTGC
	Insert = Rab7 WT, Q67L or T22N	Reverse	cgtataagcttTCAGCAACTGCAGCTTTCTG
CFP-GAI-Sac1	Insert = Sac1	Forward	tagctGAGCTCccACAGGTCCAATAGTGTACGTTC
	Insert = Sac1	Reverse	agtatGGATCCTCAGGGGAATGGCGACTTGATAG
CFP-GAI-Sac1 C392S	Insert = Sac1 C392S	Forward	GTAAGAACAAACTCTATGGATTGTTTGG
	Insert = Sac1 C392S	Reverse	AACGGAATGTTGCTCATTAACAA
mApple-Drp1	Insert = mApple	Forward	TAGTCACTCGAGATGGTGAGCAAGGGCGAGGA
	Insert = mApple	Reverse	atagctAGATCTCTTGTACAGCTCGTCCATGCC
	Vector = GFP-Drp1	Forward	atagctAGATCTGTCATGGAGGCGCTGATC
	Vector = GFP-Drp1	Reverse	tagtatCTCGAGTGACGGTCACTAAACCAGCTCT
GFP-ORPOSBP	Vector = GFP-ORP1L	Forward	tagtatACCGGTGCATACAAGTCAGGGTGTTTG
	Vector = GFP-ORP1L	Reverse	atagctCTTAAGGTAAGCAGAATGTTCTTCTATTGC
	Insert = OBSP 184-809	Forward	atagctCTTAAGAAGGCTGTGAAGATGCTGGCAGA
	Insert = OBSP 184-809	Reverse	tagtatACCGGTTCAGAAAATGTCCGGGCATGAGT
GFP-ORPOSBP HH/AA		Forward	GCTGCTCCCCCTGCTGCTGCACACCAT
		Reverse	ACTCACCTGCTCACAGAGGGATCG
iRFP-GID1-MFF	Vector = iRFP-GID1-Rab7	Forward	acggacACTAGTACTGATCATAATCAGCCATACCAC
	Vector = iRFP-GID1-Rab7	Reverse	tagtatTGTACATTGAGCACTACCACCAGCAC
	Insert = MFF	Forward	tagtatTGTACAATGAGTAAAGGAACAAGCAGTGA
	Insert = MFF	Reverse	agagcACTAGTCTAGCGGCGAAACCAGAG
GFP-Sac1-MFF	Vector = GFP-MFF	Forward	tcagcACTAGTATGAGTAAAGGAACAAGCAGTG
	Vector = GFP-MFF	Reverse	cgtatGAGCTCCTTGTACAGCTCATCCATGCC
	Insert = Sac1	Forward	tagagGAGCTCACAGGTCCAATAGTGTACGTTC
	Insert = Sac1	Reverse	tcaatACTAGTGGGGAATGGCGACTTGATAG
GFP-Sac1 C392S-MFF	Vector = GFP-MFF	Forward	tcagcACTAGTATGAGTAAAGGAACAAGCAGTG
	Vector = GFP-MFF	Reverse	cgtatGAGCTCCTTGTACAGCTCATCCATGCC
	Insert = Sac1 C392S	Forward	tagagGAGCTCACAGGTCCAATAGTGTACGTTC
	Insert = Sac1 C392S	Reverse	tcaatACTAGTGGGGAATGGCGACTTGATAG

Supplementary Table 2. List of primers used for qPCR.

All primers have been custom synthesized and purchased from Idt (Integrated DNA technologies).

Gene	Sens	Sequence
OSBPL1a	Forward	TCCGAAGAAAAAGACTGTGGTG
OSBPL1a	Reverse	CAGTTAGGCGCTGTAGGAAGC
STARD3	Forward	CGACATCTTTGTCCTGGCCT
STARD3	Reverse	GAGGAATGCACTGGACACCA
Rab7	Forward	CAGACAAGTGGCCACAAAGC
Rab7	Reverse	AAGTGCATTCCGTGCAATCG
NPC1	Forward	ACTCAGTTACATAGGGCCATCA
NPC1	Reverse	ATCGCTCTTCAGTGGCACAA
PI4K2B	Forward	GTGACAAATGTGGTAGACTAAAGGT
PI4K2B	Reverse	TCCAAACACGTACATACAGAAGA
ACTB	Forward	TGGCATCGTGATGGACTCC
ACTB	Reverse	AATGTCACGCACGATTTCCC
DNM1L	Forward	CCGGAGACCTCTCATTCTGC
DNM1L	Reverse	TCTGCTTCCACCCCATTTTCT
MFF	Forward	TCTCAGCCAACCACCTCTGA
MFF	Reverse	TGAGAGCCACTTTTGTCCCC
