# Supplementary material for: ORP1L mediated PI(4)P signaling at ER-lysosome-mitochondrion three-way contact contributes to mitochondrial division
Source: Nat Commun. 2021 Sep 9;12:5354. doi: 10.1038/s41467-021-25621-4 (PMC8429648; doi:10.1038/s41467-021-25621-4)
Supplement: Supplementary file 3 — Reporting Summary [file 41467_2021_25621_MOESM3_ESM.pdf]

nature research

Corresponding author(s): Peter K. Kim
Last updated by author(s): Aug 10, 2021

Reporting Summary

Nature Research wishes to improve the reproducibility of the work that we publish. This form provides structure for consistency and transparency in reporting. For further information on Nature Research policies, see our Editorial Policies and the Editorial Policy Checklist.

Statistics

For all statistical analyses, confirm that the following items are present in the figure legend, table legend, main text, or Methods section.

n/a	Confirmed	
☐	☒	The exact sample size (*n*) for each experimental group/condition, given as a discrete number and unit of measurement
☐	☒	A statement on whether measurements were taken from distinct samples or whether the same sample was measured repeatedly
☐	☒	The statistical test(s) used AND whether they are one- or two-sided<br>*Only common tests should be described solely by name; describe more complex techniques in the Methods section.*
☐	☒	A description of all covariates tested
☐	☒	A description of any assumptions or corrections, such as tests of normality and adjustment for multiple comparisons
☐	☒	A full description of the statistical parameters including central tendency (e.g. means) or other basic estimates (e.g. regression coefficient) AND variation (e.g. standard deviation) or associated estimates of uncertainty (e.g. confidence intervals)
☐	☒	For null hypothesis testing, the test statistic (e.g. *F*, *t*, *r*) with confidence intervals, effect sizes, degrees of freedom and *P* value noted<br>*Give P values as exact values whenever suitable.*
☒	☐	For Bayesian analysis, information on the choice of priors and Markov chain Monte Carlo settings
☒	☐	For hierarchical and complex designs, identification of the appropriate level for tests and full reporting of outcomes
☒	☐	Estimates of effect sizes (e.g. Cohen's *d*, Pearson's *r*), indicating how they were calculated

Our web collection on statistics for biologists contains articles on many of the points above.

Software and code

Policy information about availability of computer code

Data collection	Volocity 6.0, Leica Las X
Data analysis	ImageJ 1.53e, Volocity 6.0, Imaris Bitplane (Imaris 9.6), Adobe Illustrator CS6, Graphpad Prism 5 and Graphpad Prism 9

For manuscripts utilizing custom algorithms or software that are central to the research but not yet described in published literature, software must be made available to editors and reviewers. We strongly encourage code deposition in a community repository (e.g. GitHub). See the Nature Research guidelines for submitting code & software for further information.

Data

Policy information about availability of data

All manuscripts must include a data availability statement. This statement should provide the following information, where applicable:

- Accession codes, unique identifiers, or web links for publicly available datasets
- A list of figures that have associated raw data
- A description of any restrictions on data availability

All data that support the findings of this study are included in the manuscript or are available upon reasonable request to the corresponding author. Source data are provided with the manuscript.

Field-specific reporting

Please select the one below that is the best fit for your research. If you are not sure, read the appropriate sections before making your selection.

☒ Life sciences ☐ Behavioural & social sciences ☐ Ecological, evolutionary & environmental sciences

For a reference copy of the document with all sections, see nature.com/documents/nr-reporting-summary-flat.pdf

Life sciences study design

All studies must disclose on these points even when the disclosure is negative.

Sample size	No sample size calculations were performed. All sample sizes are listed in detail in the figures, figures legends and/or main text. The number of cells imaged are consistent with previous live cell imaging studies (I.e Wong et al, 2018. Nature. PMID = 29364868). This typically resulted in at least 10 cells imaged per condition per experiment from three independent experiments.
Data exclusions	In supplementary figure 5b a Grubbs' test (GraphPad) was performed which resulted in the removal of one outlier from the HH/AA condition. No other data exclusion were performed
Replication	During this study, cells were acquired from three independent experiments, except for Figures 1g-h, 1j-k, 2c, 3f-g, 4d, 5a and Supplementary Figures 1c, 2d-I, 2k, 2l, 5e that were from two independent experiments. The number of independent experiments performed is detailed in the figure's legends and in the section called "Statistics and Reproducibility" in the Methods. For representative images shown: Figures 1a, 2b, 2e, 3a, 3b, 4m, 6b, 6c, 7b, 7i and Supplementary Figures 1a, 1b, 2b, 2c, 2d, 2e, 2i, 6e, 6f are representative of two independent experiments.
Randomization	Allocation was not random as this did not apply for the live imaging experiments we performed. All cells were imaged and analyzed the same way
Blinding	Investigators were not blinded during data acquisition (as this was not possible for live imaging experiments) but acquired data were then anonymized by researchers not involved in the study and were therefore analyzed in a blind manner

Reporting for specific materials, systems and methods

We require information from authors about some types of materials, experimental systems and methods used in many studies. Here, indicate whether each material, system or method listed is relevant to your study. If you are not sure if a list item applies to your research, read the appropriate section before selecting a response.

Materials & experimental systems

n/a	Involved in the study	
☐	☒	Antibodies
☐	☒	Eukaryotic cell lines
☒	☐	Palaeontology and archaeology
☒	☐	Animals and other organisms
☒	☐	Human research participants
☒	☐	Clinical data
☒	☐	Dual use research of concern

Methods

n/a	Involved in the study	
☒	☐	ChIP-seq
☒	☐	Flow cytometry
☒	☐	MRI-based neuroimaging

Antibodies

Antibodies used	The following antibodies were used in this study: mouse anti-Lamp1 (DHSB, H4A3, dilution: 1:1000), rabbit anti-ORP1 (Abcam, ab131165, dilution: 1:1000), mouse anti-Drp1 (BD Biosciences, 611112, dilution: 1:1000), rabbit anti-VAPA (Novus Biologicals, NBP1-31237, dilution: 1:1000), rabbit-anti-VAPB (MilliporeSigma, HPA013144, dilution: 1:1000), mouse-anti GAPDH HRP conjugated (Novus Biologicals, NB300-328H, dilution: 1:10000), goat anti-Rabbit HRP (ThermoFisher, 31460, dilution: 1:10000), goat anti-Mouse HRP (Cedarlane, CLCC30007, dilution: 1:10000) and goat anti-Mouse Alexa 568 (ThermoFisher, A-11011, dilution: 1:1000).
Validation	All antibodies used have been previously validated for use in the experiments performed (immunofluorescence and/or western blot in mammalian cell lines).<br>mouse anti-Lamp1 (DHSB, H4A3, dilution: 1:1000) showed expected structures for lysosomes with immunostaining and was used in many studies for Lamp1 immunostaining (cited 36 times according to the suplier, DHSB Hybridoma bank)<br>rabbit anti-ORP1 (Abcam, ab131165, dilution: 1:1000) was validated by the supplier for human cells (abcam) and was validated using ORP1L KO CRISPR HeLa cells (Zhao et al, 2017. Cell reports. PMID = 28564600).<br>mouse anti-Drp1 (BD Biosciences, 611112, dilution: 1:1000) was shown by the supplier to probe a band at the expected molecular weight and was used in numerous studies (cited 120 times according to the supplier)<br>Rabbit anti-VAPA (Novus Biologicals, NBP1-31237, dilution: 1:1000) and rabbit-anti-VAPB (MilliporeSigma, HPA013144, dilution: 1:1000) shown bands at the expected molecular weight that strongly decreased when siRNA targeting VAPA or VAPB, respectively, were used.

mouse-anti GAPDH HRP conjugated (Novus Biologicals, NB300-328H, dilution: 1:10000) is currently discontinued but was validated for western blot for human cells by the supllier

Eukaryotic cell lines

Policy information about cell lines

Cell line source(s)	COS-7, HeLa and U-2 OS cells were obtained from ATCC. ORP1L KO HeLa cells generated using CRISPR-Cas9 technology and the ORP1L WT control were a gift from Neale Ridgway (Dalhousie University, Halifax, Canada)
Authentication	Cell lines were purchased from ATCC. ORP1L WT and KO HeLa cells were validated by western blot analysis
Mycoplasma contamination	Cells lines were regularly (every 6 months) tested negative for mycoplasma contamination.
Commonly misidentified lines (See ICLAC register)	No commonly misidentified cell lines were used
